# The association of dry eye syndrome and psychiatric disorders: a nationwide population-based cohort study

**DOI:** 10.1186/s12886-020-01395-z

**Published:** 2020-03-30

**Authors:** Chiao-Ying Liang, Wai-Man Cheang, Chun-Yuan Wang, Keng-Hung Lin, Li-Chen Wei, Yu-Yen Chen, Ying-Cheng Shen

**Affiliations:** 1 Department of Ophthalmology, Taichung Veterans General Hospital, 1650 Taiwan Boulevard Sect. 4, Xitun Dist, Taichung, 40705 Taiwan, Republic of China; 2 Graduate institute of biomedical sciences, China Medical University, Taichung, Taiwan, Republic of China; 3 Department of Ophthalmology, Taichung Tzu Chi Hospital, Taichung, Taiwan, Republic of China

**Keywords:** Dry eye syndrome, Psychiatric disorders, Bipolar, Population-based

## Abstract

**Background:**

Several previous studies reported a greater prevalence of dry eye syndrome (DES) among patients with psychiatric diseases. The aim of this study is to investigate the prevalence and risk factors of DES in patients with psychiatric disorders (PD) using nationwide population-based data in Taiwan.

**Methods:**

This population-based cohort study retrospectively identified patients with PD from 1997 to 2011. Patients with both PD and DES served as the DES cohort, and PD patients without DES comprised the non-DES cohort. PD was defined as a diagnosis of PD (ICD-9-CM 290–319) made by psychiatrists only, with at least three consecutive outpatient visits or at least one inpatient visit. DES was defined as a diagnosis of DES (ICD-9-CM 375.15) and a prescription for an eye lubricant (anatomical therapeutic chemical code, ATC code: S01XA). The main outcome measures were the prevalence of DES in these patients and associated risk factors.

**Results:**

A total of 75,650 patients with PD (3665 in the DES cohort and 71,985 in the non-DES cohort) were included in the final analysis. The majority of patients in the DES group were women (72.6%), compared the non-DES group (57.8%). The mean age of patients in the DES cohort was 62.2 ± 14.9, which was significantly older than those in the non-DES group (50.9 ± 17.5). The patients with DES had a significantly greater likelihood of having dementia, bipolar disorder, depression, and neurotic disorders. Conditional regression analyses revealed that patients with dry eye disease were more likely to have schizophrenia (OR = 1.34), bipolar disorder (OR = 1.9), depression (OR = 1.54), and neurotic disorders (OR = 1.62). In addition, patients with DES were more likely to use 1st generation anti-psychotics (OR = 1.28) and had a lower risk of using 2nd generation anti-psychotics (OR = 0.64).

**Conclusion:**

The study demonstrated that among PD patients, DES is highly prevalence in certain subtypes of PD, such as depression, bipolar disorder, and neurotic disorders, after adjusting for the comorbidities.

## Background

Dry eye syndrome (DES) is a common ocular surface disease worldwide. It is a multifactorial disease of tears and ocular surface (DEWS 2007), causing irritation, blurred vision, burning, and foreign body sensation which affect patients’ work, daily activities, quality of life, and emotions [1, 2].

Several previous epidemiological studies reported a greater prevalence of DES among patients with psychiatric diseases, such as depression, anxiety, and bipolar disorder [3–7]. However, information on the potential association between psychiatric disorders (PD) and DES is limited. Understanding of the interactions and chronology between PD, anti-psychotics, and DES is crucial for the integrated care of these patients. In the current study, we aim to investigate the prevalence and risk factors of DES in patients with psychiatric disorders using a nationwide population-based database from 1997 to 2011 in Taiwan.

## Methods

### Data source

The data were obtained from Taiwan’s National Health Insurance Research Database (NHIRD). The Taiwan’s National Health Insurance (NHI) program was established on March 1, 1995, and currently provides health care coverage for 99% of the country’s population, approximately 23 million people. In this study, we used the Longitudinal Health Insurance Database 2010 (LHID2010), a subset of the NHIRD, comprising 1,000,000 randomly selected NHI beneficiaries. Patients’ demographic data including gender, date of birth, income level, and healthcare data including diagnostic codes, drug prescriptions, and medical procedures contained in the database and are made available to researchers. To protect patients’ privacy, all personally identifiable information is encrypted prior to release. The NHI records diagnoses according to the International Classification of Diseases, 9th Revision, Clinical Modification (ICD-9-CM).

### Ethics statement

The study was approved by the Institutional Review Board of Taichung Veterans General Hospital, Taichung, Taiwan (CE13152B-6). In this study, the requirement for informed consent was waived because the patients’ original identification numbers were anonymized by encryption.

### Study population

This population-based cohort study retrospectively identified patients with PD (ICD-9-CM 290–319) from 1997 to 2011 from the LHID2010. We defined PD diagnoses as those made by psychiatrists only, with at least three consecutive outpatient visits or at least one inpatient visit. Patients with both PD and DES (ICD-9-CM 375.15) served as the DES cohort, and PD patients without DES comprised the non-DES cohort. DES was defined as a diagnosis of DES and a prescription for an eye lubricant (anatomical therapeutic chemical code, ATC code: S01XA). Subjects who had a diagnosis of DES prior to the diagnosis of PD were excluded from this study. In addition, we excluded patients younger than 18 years and any PD diagnosis related to children or infants (ICD-9-CM 299, 312–316). Moreover, patients with rheumatoid arthritis (RA), systemic lupus erythematosus (SLE), and Sjogren’s syndrome (SS) patients were also excluded.

### Main measure outcomes

The main outcome measures were the prevalence of DES in PD patients and associated risk factors.

### Statistical analysis

The statistical analyses were conducted using the SAS 9.3 statistical package (SAS Institute Inc., NC, USA). We used Pearson’s Chi-square tests to compare the prevalence rates of different PD among patients with and without DES. In addition, the odds ratio (OR) and 95% confidence interval (CI) for gender, age, and each subtype of the PD were conducted to calculate the risk among patients with and without DES. A *p*-value < 0.05 in 2-tailed tests was defined as significant.

## Results

**Fig. 1:**
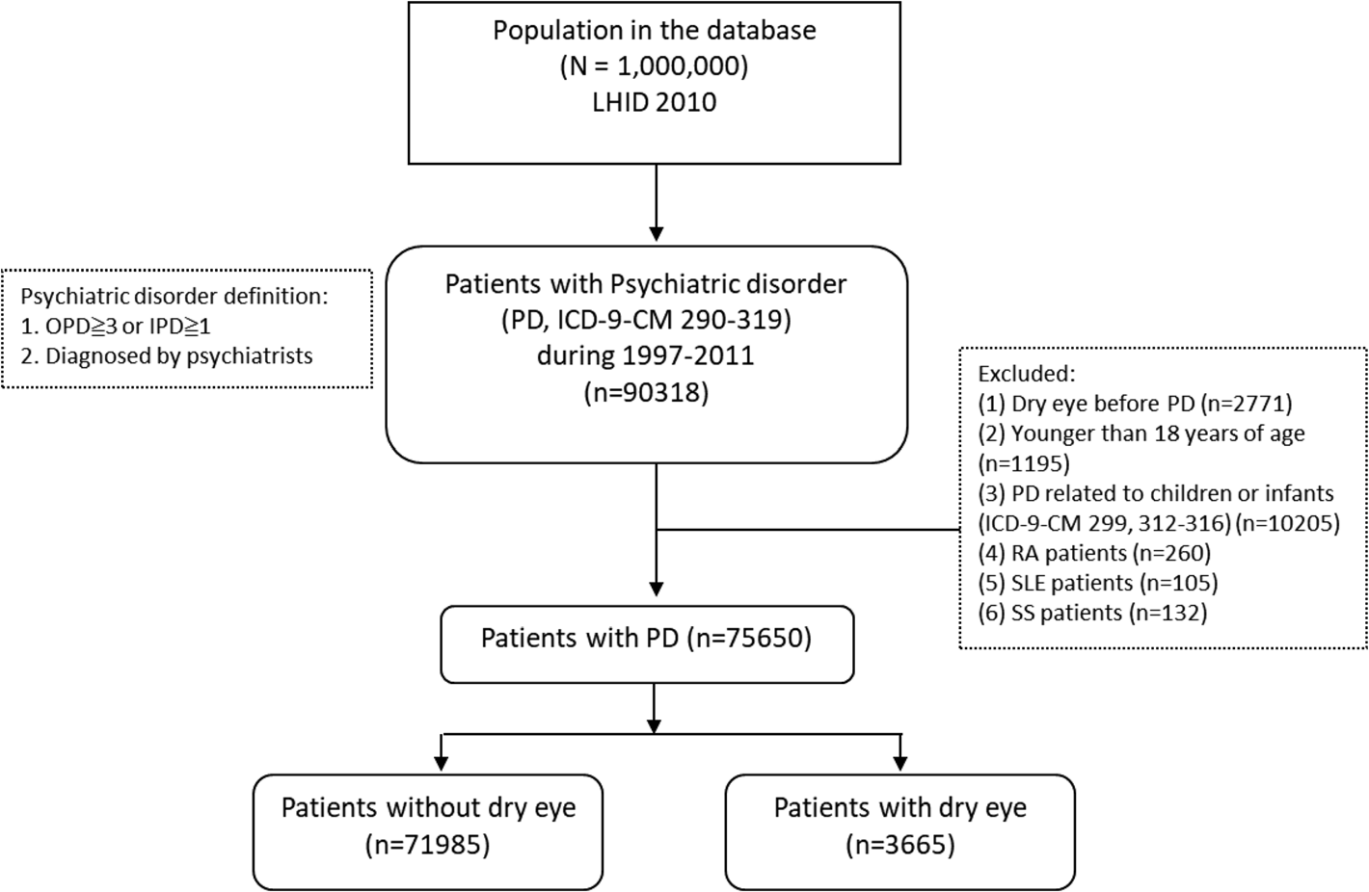
Flowchart of the study sample selection from the National Health Insurance Research Database

Among the 1,000,000 subjects in the database, 90,318 patients were identified as being diagnosed with a PD (Fig. 1). We excluded 14,668 patients who had a diagnosis of DES prior to the diagnosis of PD (*n* = 2771), were younger than 18 years (*n* = 1195), had a PD diagnosis related to children or infants (*n* = 10,205), or had a diagnosis of RA (*n* = 260), SLE (*n* = 105), or SS (*n* = 132). The remaining 75,650 patients with PD (3665 in the DES cohort and 71,985 in the non-DES cohort) were included in the final analysis.

The baseline characteristics of the patients in the two groups are compared in Table 1. The majority of patients in the DES group were women (72.6%). The mean age (±standard deviation) of the patients in the DES cohort was 62.2 ± 14.9, which was significantly older than that of the non-DES group (50.9 ± 17.5).

**Table 1:** Baseline characteristics of PD patients with and without dry eye for the period 1997–2011

	Dry Eye Syndrome (DES)				
	No *n* = 71,985		Yes *n* = 3665		
Variables	n	(%)	n	(%)	*p*-value
**Gender**					< 0.001
Women	41,594	(57.8)	2660	(72.6)	
Men	30,391	(42.2)	1005	(27.4)	
**Age, years**					< 0.001
Mean ± SD	50.9 ± 17.5		62.2 ± 14.9		< 0.001
< 30	8402	(11.7)	77	(2.1)	
30–39	13,367	(18.6)	227	(6.2)	
40–49	15,213	(21.1)	494	(13.5)	
50–59	14,499	(20.1)	760	(20.7)	
60–69	8837	(12.3)	891	(24.3)	
≧70	11,667	(16.2)	1216	(33.2)	

The prevalence rates of the different diagnoses of PD in patients with and without DES are shown in Table 2. The patients with DES had significantly greater prevalence rates of dementia, bipolar disorder, depression, and neurotic disorders. In contrast, prevalence rates of drug or alcoholic psychosis, schizophrenia, and psychiatric retardation were lower in DES patients. No significant differences in the prevalence rates of paranoid states and obsessive-compulsive disorders were found between patients in the DES and non-DES cohorts.

**Table 2:** Psychiatric disorder conditions as defined by ICD-9-CM in PD patients with and without dry eye for the period 1997–2011

		Dry Eye Syndrome				
		No *n* = 71,985		Yes *n* = 3665		
**Psychiatric disorder condition**	**(ICD-9-CM)**	n	(%)	n	(%)	*p*-value
Dementia	(290, 331.0, 331.2)	7426	(10.3)	534	(14.6)	< 0.001
Alcoholic psychoses	(291)	1707	(2.4)	35	(1.0)	< 0.001
Drug psychoses	(292)	1339	(1.9)	38	(1.0)	< 0.001
Schizophrenia	(295)	4242	(5.9)	151	(4.1)	< 0.001
Bipolar disorder	(296)	2968	(4.1)	267	(7.3)	< 0.001
Major depression	(296.2, 296.3)	17,001	(23.6)	1180	(32.2)	< 0.001
Paranoid states	(297)	1822	(2.5)	90	(2.5)	0.777
Neurotic disorders	(300)	59,528	(82.7)	3400	(92.8)	< 0.001
Obsessive-compulsive disorders	(300.3)	2059	(2.9)	109	(3.0)	0.687
Minor depression	(300.4, 309.0, 309.1, 311)	38,542	(53.5)	2382	(65.0)	< 0.001
Mild mental retardation	(317)	1136	(1.6)	17	(0.5)	< 0.001
Other specified mental retardation	(318)	949	(1.3)	4	(0.1)	< 0.001
Unspecified mental retardation	(319)	1266	(1.8)	16	(0.4)	< 0.001

Table 3 shows the crude and adjusted OR and 95% CI for each of the PD, comparing patients with and without DES.

**Table 3:** A comparison of baseline characteristics of dry eye and non-dry eye patients based on PD conditions in univariate and multivariate models

Variables	Univariate model			Multivariate model^b^		
	OR^a^	(95% CI)	*p*-value	OR	(95% CI)	*p*-value
**Gender**						
Women	1.00	(reference)		1.00	(reference)	
Men	0.52	(0.48–0.56)	< 0.001	0.60	(0.56–0.65)	< 0.001
**Age, years**						
< 40	1.00	(reference)		1.00	(reference)	
40–49	1.85	(1.43–2.40)	< 0.001	1.38	(1.06–1.80)	< 0.001
50–59	3.54	(2.78–4.51)	0.001	2.17	(1.70–2.78)	0.000
60–69	5.72	(4.52–7.24)	< 0.001	2.97	(2.33–3.79)	0.001
70–79	11.00	(8.70–13.9)	< 0.001	5.24	(4.10–6.72)	< 0.001
≧80	11.37	(9.01–14.3)	< 0.001	6.42	(4.98–8.27)	< 0.001
**Psychiatric disorder (Yes vs. No)**						
Dementia	1.48	(1.35–1.63)	< 0.001	0.73	(0.65–0.82)	< 0.001
Alcoholic psychoses	0.40	(0.28–0.56)	< 0.001	0.59	(0.42–0.83)	0.003
Drug psychoses	0.55	(0.40–0.76)	< 0.001	0.85	(0.61–1.18)	0.332
Schizophrenia	0.69	(0.58–0.81)	< 0.001	1.34	(1.11–1.62)	0.002
Bipolar disorder	1.83	(1.60–2.08)	< 0.001	1.90	(1.65–2.19)	< 0.001
Major depression	1.54	(1.43–1.65)	< 0.001	–	–	–
Paranoid states	0.97	(0.78–1.20)	0.779	1.01	(0.81–1.27)	0.928
Neurotic disorders	2.68	(2.36–3.05)	< 0.001	1.62	(1.42–1.85)	< 0.001
Obsessive-compulsive disorders	1.04	(0.86–1.27)	0.682	–	–	–
Minor depression	1.61	(1.50–1.73)	< 0.001	–	–	–
Mild mental retardation	0.29	(0.18–0.47)	< 0.001	1.15	(0.69–1.93)	0.597
Other specified mental retardation	0.08	(0.03–0.22)	< 0.001	0.25	(0.09–0.70)	0.008
Unspecified mental retardation	0.24	(0.15–0.40	< 0.001	0.84	(0.49–1.43)	0.519
**Anti-psychotics**						
None	1.00	(reference)		1.00	(reference)	
First generation	2.06	(1.87–2.27)	< 0.001	1.28	(1.15–1.41)	< 0.001
Second generation	0.65	(0.44–0.94)	< 0.001	0.64	(0.44–0.95)	0.005
Both	1.61	(1.42–1.81)	< 0.001	1.07	(0.92–1.23)	0.118

^a^OR, odds ratio

^b^adjusted for all variables in table

Conditional regression analyses conditioned on gender and age revealed that compared to patients without DES, patients with DES were more likely to have schizophrenia (OR = 1.34), bipolar disorder (OR = 1.9), depression (OR = 1.54), and neurotic disorders (OR = 1.62). In addition, patients with DES were more likely to use 1st generation anti-psychotics (OR = 1.28) and were less likely to use 2nd generation anti-psychotics (OR = 0.64).

## Discussion

The aim of the current study was to use a nationwide population-based database to evaluate the prevalence of DES in patients with PD and to investigate the associated risk factors for the period 1997 to 2011. We found the prevalence of DES in adult patients with PD was 4.84% (*n* = 3665/75650).

Among the patients with PD in our study, female gender, older age, and a number of medical comorbidities were associated with higher risk of DES after adjustment, and the result is consistent with prior studies [8, 9]. Furthermore, patients with DES had significantly greater risks of schizophrenia (OR = 1.34), bipolar disease (OR = 1.90), depression (OR = 1.54), sleeping disturbance/ insomnia (OR = 1.19), and neurotic disorders (OR = 1.24), including anxiety (OR = 1.34), than those without DES. However, prevalence of DES was significantly lower in patients with dementia (OR = 0.73) and mental retardation (OR = 0.25).

Several previous studies demonstrated greater prevalence of DES in people with depression [3–5, 8, 9]. Furthermore, the severity of DES had a greater impact on the depressive symptoms compared with other psychosomatic symptoms [10]. Several mechanisms may explain the association between depression and DES [11–13]. First, we believe that inflammation may play a key role in the pathogenesis of both depression and DES. In patients with DES, increased production of inflammatory cytokines was found in the tears and conjunctiva, including TNF-a, IFN-c, IL-1b, IL-2, IL-6, and IL-8.47–51 [14–16]. Depressive patients were also reported to have higher levels of inflammatory cytokines and neuropeptides in the blood [15–17]. These cytokines and neuropeptides may simultaneously lead to ocular surface inflammation and exacerbation of negative moods. Second, many previous studies reported depressive patients have a lower threshold of pain perception and often complaint worse dry eye symptom compare with patients without depression. “Neuropathic pain” caused by neural dysfunction plays a role in the unreasonable chronic pain in patients with DES and depression [18]. Third, selective serotonin reuptake inhibitors (SSRIs) are a class of antidepressant that have been reported to be significantly associated with DES. It is possible that SSRIs cause an anticholinergic side effect whereby altered serotonin levels affect the sensitivity thresholds of corneal nerves [19]. Further study on the correlation between SSRIs and DES is necessary.

Our study also found that anxiety disorder was correlated with DES, which supports the findings of two previously reported case-control studies [4, 20]. Furthermore, Wen et al. found three significant independent predictors of DES in patients with anxiety, including older age, duration of PD, and the use of an SSRI [4].

There was a significantly increased risk of DES in patients with bipolar disorder (OR = 1.55). Dibajnia et al. showed significant decreased tear break up time in patients with bipolar disorder treated with either lithium carbonate or sodium valproate [5]. However, the mechanism of this pharmacologic effect is not clear.

Older age has been shown to be associated with increased risk of both DES and dementia [1]. Previous studies demonstrated that patients with dementia have a greater risk of developing dry eye and Sjögren’s syndrome [21, 22]. However, we found a decreased risk of DES in dementia patients in our multivariate models. This is the first large-scale study to find this association. We believe the risk might have been underestimated because dementia patients may be less likely to report symptoms due to the decline in cognitive function and communication skills.

A major strength of this study was the use of a large cohort study to investigate the association between DES and PD using data from the National Health Insurance Research Database (NHIRD). This database covers approximately 99% of the country’s residents. However, there were several limitations in this study. First, it was a retrospective study and detailed information are not available in the NHIRD, such as socioeconomic status, severity of disease, and laboratory results. Second, we used strict exclusion criteria and definitions for the diagnosis of DES. Only patients with both ICD-9-CM diagnosis code 375.15 and an anatomical therapeutic chemical code for ophthalmological lubricants (ATC code: S01XA) were included in the analysis. This selection bias might have resulted in an underestimation of the prevalence of DES in psychiatric patients, because patients with only mild dry eye symptoms may not need to use any medication. In addition, some patients may seek other medications that are not available on the NHI system.

## Conclusion

This nationwide population-based cohort study demonstrated that among PD patients, DES is highly prevalence in certain subtypes of PD, such as depression, bipolar disorder, and neurotic disorders, after adjusting for the comorbidities.

## Data Availability

The datasets used and/or analysed during the current study are available from the corresponding author on reasonable request.

## Abbreviations

ATC: Anatomical therapeutic chemical code
CI: Confidence Interval
DEWS: Dry Eye Work Shop
DES: Dry Eye Syndrome
ICD-9-CM: International Classification of Diseases, 9th Revision, Clinical Modification
IL: Interleukins
LHID: Longitudinal Health Insurance Database
NHI: National Health Insurance
NHIRD: National Health Insurance Research Database
OR: Odds Ratio
PD: Psychiatric Disorders
RA: Rheumatoid Arthritis
SLE: Systemic Lupus Erythematosus
SS: Sjogren’s Syndrome
SSRIs: Selective Serotonin Reuptake Inhibitors
TNF: Tumor Necrosis Factor
